# Acta Veterinaria Scandinavica – now an open access journal

**DOI:** 10.1186/1751-0147-48-1

**Published:** 2006-05-26

**Authors:** Mats Forsberg

**Affiliations:** 1 Professor em., Faculty of Veterinary Medicine and Animal Science, Swedish University of Agricultural Sciences, Uppsala, Sweden

Welcome to *Acta Veterinaria Scandinavica *[1]! *Acta Veterinaria Scandinavica *was founded in 1959 as a traditional print journal, but has now taken the novel step of moving to the open access model already used successfully by our publisher, BioMed Central [2]. *Acta Veterinaria Scandinavica *will be freely available online, and will continue to publish articles that encompass all aspects of veterinary research and medicine of domestic animals and wildlife. One of our aims is to close the gap between research in veterinary medicine and animal science, so we also welcome manuscripts from related fields within biology.

Why 'open access'?

Traditionally, readers pay to access research articles, either through subscriptions or by paying a fee each time they download an article. Escalating journal subscription charges have resulted in libraries subscribing to fewer journals [3], and the range of research available to readers is therefore increasingly limited. Having to pay to access research articles limits how many can read, use and cite them. Often only the title, keywords, and abstract are available for free. As a consequence, many researchers read only the abstract of the paper they are interested in, rather than the full article.

From a scientific point of view, a far better strategy is to offer all readers unrestricted access to the full articles. Such a strategy has at least three benefits. First, authors are assured that their work is disseminated to the widest possible audience and the authors are free to reproduce and distribute their work. Second, the information available to researchers will not be limited by their library's budget. Third, a country's economy will not influence its scientists' ability to access research.

To encourage submissions, *Acta Veterinaria Scandinavica *will provide a quick and impartial closed peer-review process. The Editor-in-Chief/Deputy Editors will first examine manuscripts, and submissions meeting the journal's standards will then result in appropriate reviews (the Editorial Board of *Acta Veterinaria Scandinavica *[4] includes 25 associate editors). Authors and reviewers will be required to complete a declaration of competing interests. Based on referee reports the Editor-in-Chief/Deputy Editors will make a final decision regarding acceptance of the manuscripts. If accepted, manuscripts will be published online immediately, which will be followed by PubMed listing.

To enable the journal to make all of its content open access, *Acta Veterinaria Scandinavica *will levy an article-processing charge (APC) for each manuscript accepted after peer review (payable on acceptance). No charge is made for articles that are rejected after peer review. However, many colleagues will find that their institutions or funding agencies will cover the costs, or support publication at a discounted rate [5,6].

I encourage colleagues to carefully weigh the scientific and financial arguments for open access and to submit articles to *Acta Veterinaria Scandinavica*. The scientific value of an article published in *Acta Veterinaria Scandinavica *can then be subject to the evaluation of the *entire *scientific community.

## References

[B1] Acta Veterinaria Scandinavica http://www.actavetscand.com/.

[B2] BioMed Central Open Access Charter. http://www.biomedcentral.com/info/about/charter.

[B3] Mayor S (2003). Libraries face higher costs for academic journals. BMJ.

[B4] Acta Veterinaria Scandinavica Editorial Board http://www.actavetscand.com/edboard.

[B5] BioMed Central Institutional Membership http://www.biomedcentral.com/inst/.

[B6] Funding agencies that explicitly allow direct use of their grants to cover article- processing charges http://www.biomedcentral.com/info/authors/apcfaq#grants.

